# Targeting TRPV1 channels in desensitized neural afferent pathways may help mitigate pain and lower urinary tract symptoms caused by prostatitis

**DOI:** 10.3389/fphar.2025.1541684

**Published:** 2025-02-25

**Authors:** ZhiPeng Jiang, AnGuo Li, Wen Luo, XiKe Luo, DeCan Liang, Jing Li, KaiHua Tang, Lei Liu, ZongMin Long, Ruiyu Miao, Lei Jian, XiaoChuan Gong, ShangJun Li, Yang Zhang, ChaoYong Yuan

**Affiliations:** ^1^ Department of Urology, The Third Affiliated Hospital of Zunyi Medical University (The First People’s Hospital of Zunyi), Zunyi, China; ^2^ Scientific Research Center, The Third Affiliated Hospital of Zunvi Medical University (The First People’s Hospital of Zunyi), Zunyi, China

**Keywords:** prostatitis, resiniferatoxin, cross-organ sensitization, desensitization, therapy

## Abstract

Chronic prostatitis/chronic pelvic pain syndrome (CPPS/CP) is a prevalent urinary disorder primarily characterized by pelvic pain and discomfort, bladder dysfunction, and sexual dysfunction. Currently, there is no effective method to alleviate the pain and lower urinary tract symptoms associated with chronic prostatitis. Resiniferatoxin (RTX), a highly potent TRPV1 receptor agonist, functions as a molecular analgesic by desensitizing TRPV1-expressing nerves. While RTX has demonstrated significant efficacy in treating various conditions, research on its application for prostatitis remains lacking. Consequently, we established a prostatitis model to investigate whether RTX could alleviate the symptoms associated with this condition. Our observations indicated that both low-dose (200 μg/kg) and high-dose (300 μg/kg) RTX effectively relieved pain and lower urinary tract symptoms caused by prostatitis. We noted that RTX reduced the expression of central pain-inducing substance P by reducing TRPV1 expression in the dorsal root ganglia (DRG), thereby mitigating pain. RTX also desensitizes bladder nerves by reducing TRPV1 expression in the bladder, which helps alleviate lower urinary tract symptoms caused by prostatitis. Collectively, these findings suggest that RTX may serve as a viable treatment option for alleviating prostatitis-induced pain and lower urinary tract symptoms through neural desensitization, presenting a promising therapeutic avenue for patients with prostatitis.

## 1 Introduction

Chronic prostatitis/chronic pelvic pain syndrome (CPPS/CP) is a prevalent condition affecting the urinary system, primarily characterized by pain and discomfort in the pelvic region, urinary dysfunction, and sexual insufficiency. The pathogenesis of CPPS/CP can be elucidated by the theory of cross-organ sensitization. The theory posits that painful stimuli originating from one affected pelvic organ may be transmitted to adjacent healthy organs (Qin et al., 2005; Malykhina, 2007), This theory is supported by a variety of animal experiments (Xia et al., 2012; Schwartz et al., 2016; Park et al., 2018; Funahashi et al., 2019; Ni et al., 2019; Aydogdu et al., 2021). Consequently, potential treatments for chronic prostatitis may be further investigated in light of this theory.

Current treatments for prostatitis primarily encompass pharmacological approaches, including antibiotics, α-blockers, anti-inflammatory agents, immunomodulators, phytotherapy, and neuromodulators, as well as non-pharmacological interventions such as pelvic floor physical therapy, acupuncture, electroacupuncture, shock wave therapy, and local heat therapy (Pena et al., 2021). Although some patients have experienced relief from prostatitis symptoms following the aforementioned treatments, many continue to suffer from persistent symptoms. Therefore, there is a pressing need to explore new therapeutic options for individuals with chronic prostatitis.

Resiniferatoxin (RTX), a TRPV1(transient receptor potential vanilloid 1) agonist, is widely utilized as a molecular analgesic in the treatment of various diseases and has demonstrated remarkable efficacy (Brown, 2016; Szallasi, 2023). RTX exerts its pharmacological effects by acting on TRPV1 targets in both neural and non-neural tissues (Iadarola and Gonnella, 2013; Bevan et al., 2014). Notably, a significant expression of TRPV1 receptors has been observed in bladder tissue and bladder sensory nerves (Aghazadeh Tabrizi et al., 2017). Clinical studies have demonstrated that the intravesical application of RTX can be an effective treatment for overactive bladder, among other conditions (Szallasi, 2023). Secondly, RTX can also desensitize nerves by interacting with the nervous system, thereby producing an analgesic effect (Iadarola and Gonnella, 2013). Therefore, by combining the pathogenesis of chronic prostatitis (cross-organ sensitization) and the unique pharmacological effects of RTX (neural desensitization), it is possible to further explore the therapeutic effect of RTX on chronic prostatitis diseases.

This study simulated the pain and lower urinary tract symptoms associated with chronic prostatitis by injecting formalin into the prostate of rats. Subsequently, different doses of RTX were administered subcutaneously to the rats, and their urination and pain responses were observed. Additionally, we assessed the expression of TRPV1 and its related substances within the cross-organ cross-sensitization pathway of prostatitis to gain further insights into the specific mechanism by which RTX may treat chronic prostatitis.

## 2 Materials and methods

### 2.1 Animals and groups

Twenty male Sprague–Dawley rats were purchased from Jiangsu Huachuang Sino Pharmaceutical Technology Co., Ltd. Twenty 9-week-old SD rats were randomly divided into 4 groups after being conventionally fed for 1 week: control group (n = 5): an intraprostatic injection of 50 µL of 0.9% NaCl solution on the 1st day and a single subcutaneous injection of 1 mL of RTX carrier solution (10% DMSO, 40% PEG300, 5% Tween 80, 45% normal saline) on the 20th day; prostatitis group (n = 5): an intraprostatic injection of 50 µL of 5% formalin on the 1st day and a single subcutaneous injection of 1 mL of RTX carrier solution on the 20th day; low-dose treatment group (n = 5): a single subcutaneous injection of 1 mL of 200 μg/kg RTX (Glpbio, Montclair, CA, United States) on the 20th day of induced prostatic inflammation; and high-dose treatment group (n = 5): a single subcutaneous injection of 1 mL of 300 μg/kg RTX on the 20th day of induced prostatic inflammation (Szallasi et al., 1999; Kun et al., 2012; Funahashi et al., 2019). 1mL of RTX solution was injected into the dorsal neck (0.4 mL) and bilateral inguinal region (0.3 mL each) of SD rats. To prevent drug leakage outside the prostate during injection, it is recommended to keep the needle at the injection site for approximately 30 s and then wipe the site with a cotton swab (Aydogdu et al., 2021). All rats were sacrificed on day 30 to collect prostate, bladder, spinal cord and dorsal root ganglion (bilateral L6 to S1) tissues. This study was approved by the Ethics Committee of the first People’s Hospital of Zunyi (2024-2-681).

### 2.2 Assessment of urinary behavior by urine spot test

In the urine spot experiment, the rats were placed in a cage covered with No. 4 filter paper (no autofluorescence) with a thickness of 0.7 mm and allowed to urinate freely for 4 h on days 0, 20, and 30. Subsequently, collect the urine-stained filter paper, observe it with a UV analyzer, image it under 365 nm UV light, and then use ImageJ analysis to perform statistical analysis on the urine-stained image (Luo et al., 2023). Subsequently, our previous urine standard curve showed that 1 mm is approximately equal to 0.25 μL of urine (Luo et al., 2024), and the rat urine output was calculated through this standard curve.

### 2.3 Assessment of chronic pelvic pain by von Frey filament

Tactile allodynia and hyperalgesia were measured using von Frey filaments. Rats in both groups were evaluated on days 0, 20, 22, 24, 26 and 28. The specific method is as follows (Okamoto et al., 2018; Zhang et al., 2019a): the rat was placed in a transparent plastic chamber with a stainless steel wire grid and allowed to adapt for 30 min. Subsequently, Von Frey fibers weighing 1 g, 2 g, 4 g, 8 g, 15 g, and 26 g were administered to vertically stimulate the rat’s pubic area or scrotum sequentially. Each stimulation lasted 2 s and was repeated with the same intensity after a 5-second interval. Each intensity level was applied 20 times, with a 5-minute interval between different intensities. In this experiment, positive reactions to Von Frey fiber stimulation in animal models included sharp contraction of the abdomen, immediate licking or scratching of the stimulated area, and jumping. Response frequency was determined as the percentage of positive responses, with data presented as mean ± standard deviation.

### 2.4 Histological analyses

All rats were sacrificed after anesthesia, and the prostate and bladder tissues were removed. The excised tissue was fixed in 4% paraformaldehyde (Biosharp; cat.BL539A) for 24 h and subsequently embedded in paraffin. The prostate and bladder tissues were then sliced into 5 μm thick sections, stained with hematoxylin-eosin (HE), and examined under a light microscope to assess morphological changes in the prostate and bladder.

### 2.5 Western blotting analyses

Rat L6-S1 dorsal root ganglion (DRG) and spinal cord were collected, frozen at −80°C, and homogenized. Proteins were extracted from rat spinal cord and DRG respectively, and the protein concentration in each tissue was measured using a BCA kit (Beyotime; cat.P0012S). Subsequently, the extracted protein was mixed with buffer at a ratio of 4:1, added to boiling water, and kept in a boiling water bath for 10 min. Denatured proteins are then loaded onto SDS-PAGE for electrophoresis. After electrophoresis, the protein is transferred to the membrane, and blocking is performed after the membrane transfer is completed. The DRG protein is hybridized with the primary antibody TRPV1 (1:2000; Bioss; cat.bs-23927R) and the secondary antibody (1:1000; Beyotime; cat. A0208). Additionally, the spinal protein was hybridized with the primary antibody substance P (SP) (1:2000; Affinity; cat. DF7522) and the secondary antibody (1:1000; Beyotime; cat. A0208). Image-pro plus (IPP) software was used to measure the optical density value of the protein band and compare the expression levels of the two groups of proteins.

### 2.6 Immunofluorescence analysis

The prepared prostate and bladder tissue sections were washed with TBST buffer (Qisai Biotechnology Co., Ltd, cat.BT-P308), followed by the addition of blocking serum. Primary antibodies PGP9.5 (1:200; Abcam; cat.14730-1-AP) and TRPV1(1:500; Abcam; cat. Ab203103) (overnight at 4°C) and HRP-conjugated secondary antibodies (PGP 9.5: 1:100; BOSTER; cat.BA1032. TRPV1: 1:100; BOSTER; cat.BA1101) (37°C for 30 min) were added for incubation, followed by a drop of Cy3 tyramide (AAT, cat.11065) (37°C for 30 min). Finally, DAPI (Beyotime; cat.C1002) nuclear staining was performed, and the slides were sealed with a mounting solution containing an anti-fluorescence quench agent. Finally, the images were observed and taken under a fluorescence microscope. Utilize ImageJ software to measure the immunofluorescence intensity of each image at a magnification of ×200. Each group was repeated more than three times, and calculate the average intensity value.

### 2.7 Measurement of proinflammatory cytokines in the prostate

The levels of tumor necrosis factor-α (TNF-α) and interferon-γ (IFN-γ) in prostate tissue were measured using the rat TNF-α ELISA kit (Biofine; FU-D1393) and rat IFN-γ ELISA kit (Biofine; FU-D1106), respectively. All samples were processed in strict accordance with the manufacturer’s instructions.

### 2.8 Statistical analysis

All values were expressed as mean ± SD, Differences between groups were conducted using a one-factor ANOVA. P < 0.5 was considered as a statistically significant difference. All data were analyzed using GraphPad Prism version 10.00.

## 3 Results

### 3.1 Analysis of inflammation in the prostate and bladder

**FIGURE 1:**
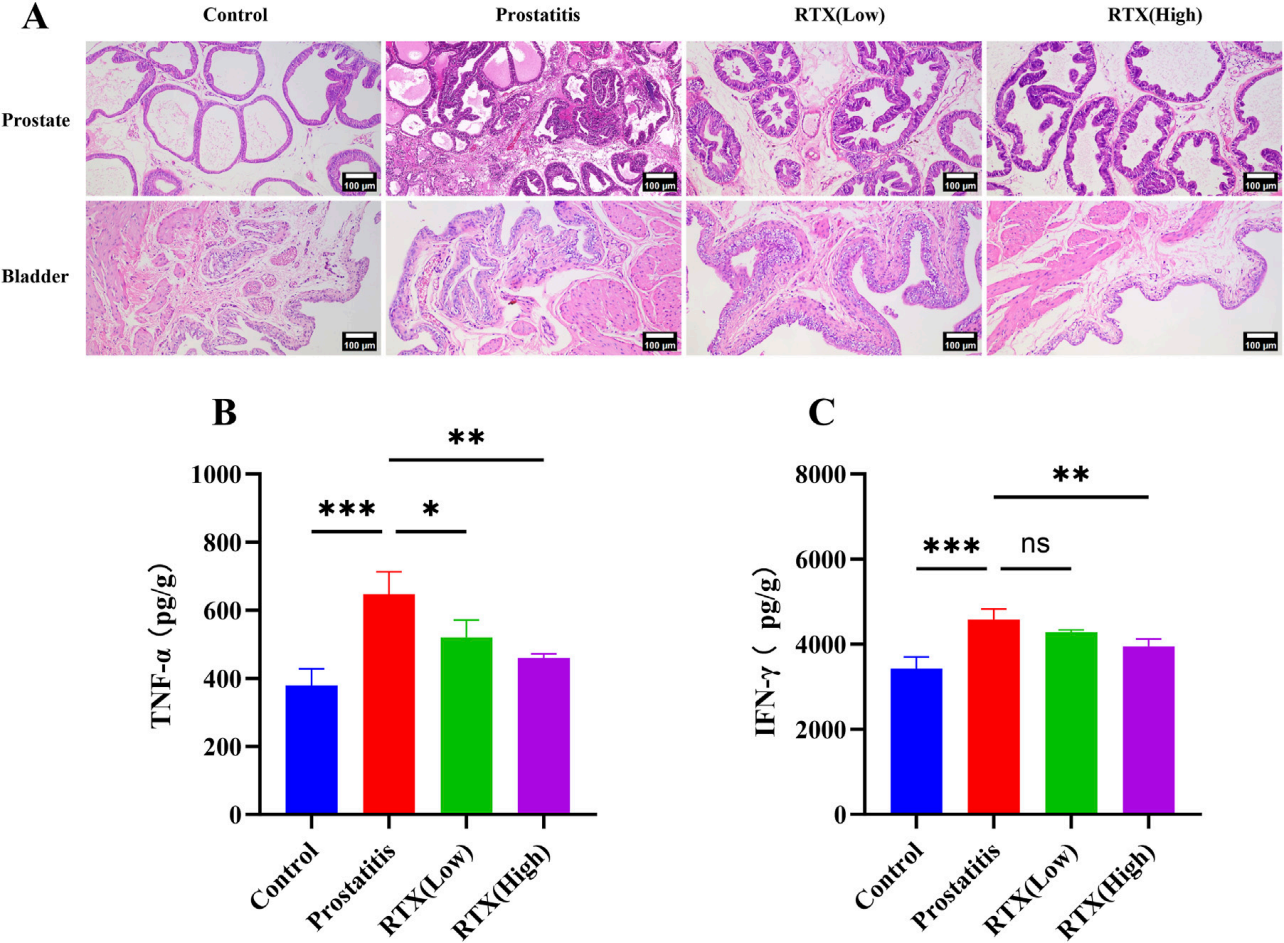
Inflammation of prostate and bladder. **(A)** Histopathological changes in the prostate and bladder. Expression of proinflammatory cytokines TNF-α **(B)** and IFN-γ **(C)** in prostate tissue. Differences between groups were compared by a one-factor ANOVA. Data are expressed as mean ± SD. *Significant difference compared with the Control group, *p < 0.05; **p < 0.01; ***p < 0.001; “ns” indicates p > 0.05. Abbreviations: TNF-α, tumor necrosis factor-α; IFN-γ, interferon-γ.

In the examination of prostate histopathology, we noted a significant increase in the distribution of inflammatory cells within the prostate stroma in the prostate inflammation group compared to the control group. Additionally, the acinar structure exhibited varying degrees of destruction, characterized by differing shapes and sizes. Both the high-dose and low-dose treatment groups demonstrated a reduction in prostatic inflammation, with only minimal inflammatory cell infiltration and irregular acinar structures observed (Figure 1A). To further elucidate the alterations in associated inflammatory factors within the prostate, we assessed the expression levels of the proinflammatory cytokines TNF-α and IFN-γ (Wang et al., 2018; 2023). Our results indicate that, in comparison to the prostatitis group, the expression levels of the proinflammatory factors TNF-α and IFN-γ exhibited varying degrees of reduction following treatment with RTX at different dosages. (Figures 1B, C). Notably, no inflammatory cell infiltration was detected in the bladder tissue pathology (Figure 1)

### 3.2 Assessment of urination behavior

**FIGURE 2:**
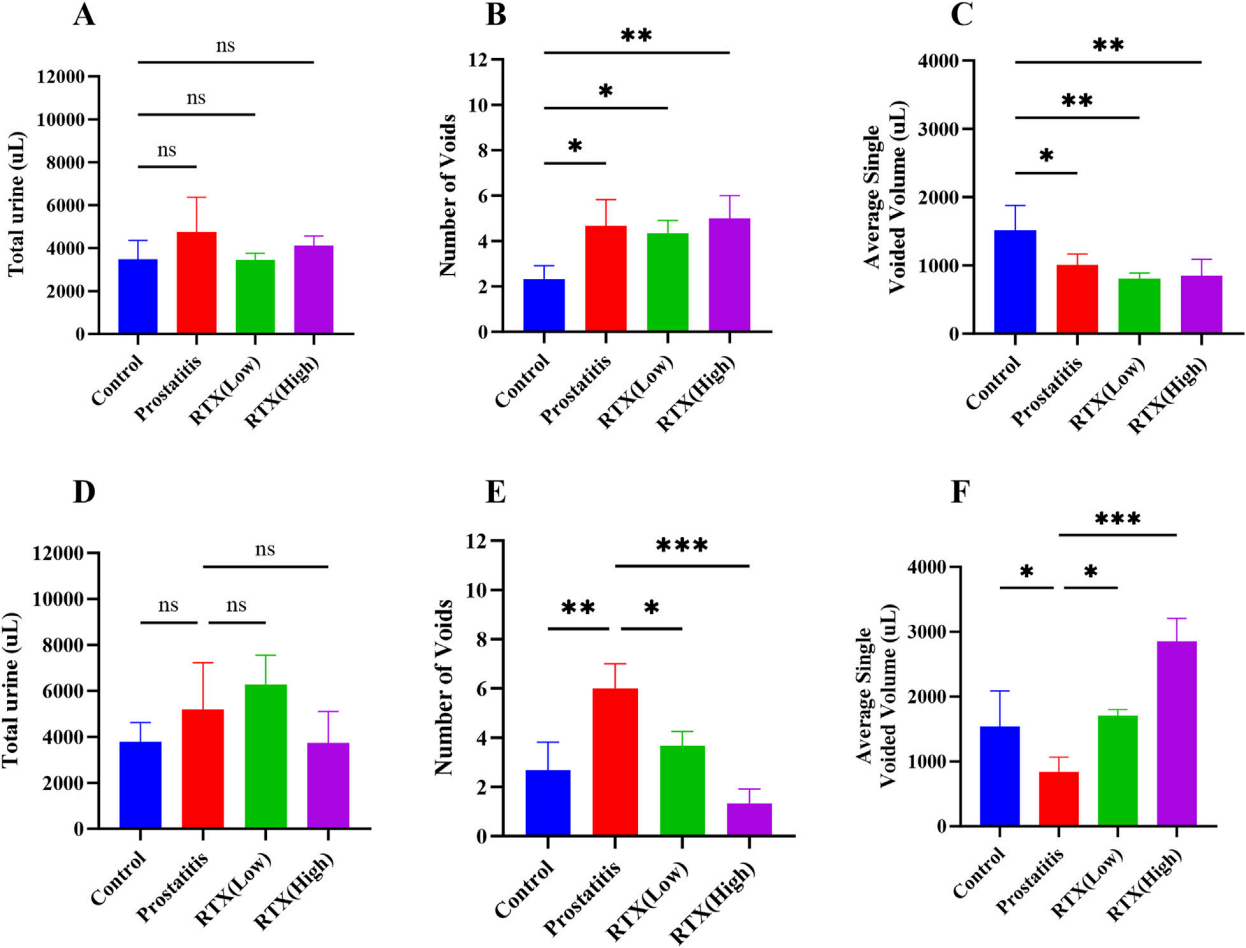
Analysis of urination behavior. Total 4-h urine volume on day 20 **(A)** and day 30 **(D)**. Quantification of the number of micturition spots on day 20 **(B)** and day 30 **(E)**. Average single urine volume on day 20 **(C)** and day 30 **(F)**. Differences between groups were compared by a one-factor ANOVA. Data are expressed as mean ± SD. *Significant difference compared with the Control group or prostatitis group, *p < 0.05; **p < 0.01; ***p < 0.001; ****p < 0.0001; “ns” indicates p > 0.05.

To evaluate the changes in the urinary behavior of rats with prostatitis treated with RTX, we assessed their urinary behavior on days 20 (Figures 2A–C) and 30 (Figures 2D–F). Our analysis revealed that, on the 20th day of modeling, the total urinary volume in the other experimental groups did not change significantly compared to the control group (Figure 2A). However, we observed an increase in the frequency of urination (Figure 2B) and a decrease in the single urinary volume (Figure 2C), which aligns with our previous findings (Luo et al., 2024). Following treatment with RTX at varying doses over a period of 10 days, no significant changes were noted in the total urinary volume across the groups when compared to the prostatitis group (Figure 2D). In contrast, we recorded a decrease in the frequency of urination (Figure 2E) and an increase in the single urinary volume (Figure 2F). These findings indicate that RTX treatment at different doses can alleviate bladder overactivity associated with prostatitis.

### 3.3 Von Frey filament in behavioral testing

**FIGURE 3:**
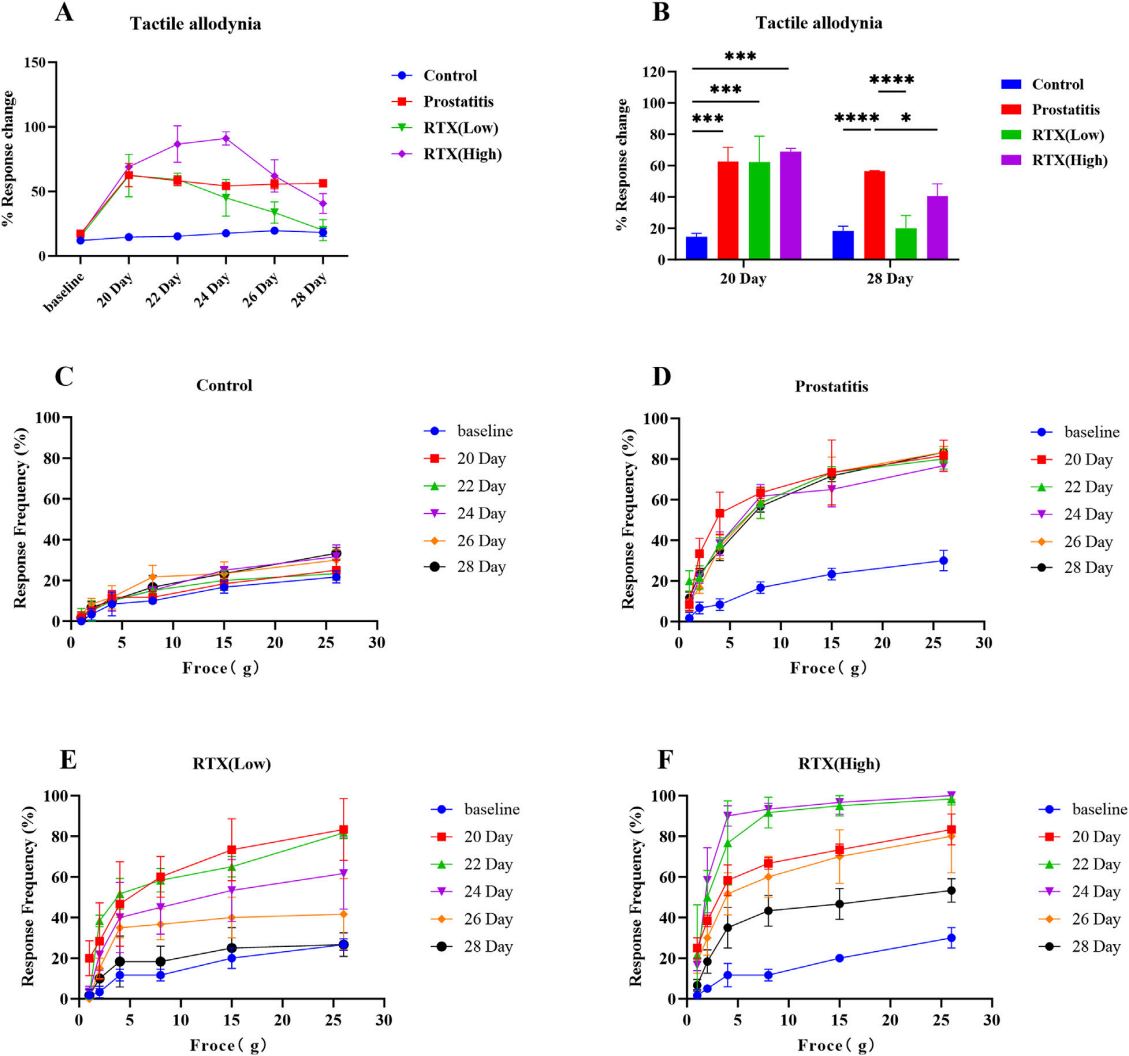
Abnormal pelvic pain in rats with prostatitis. **(A)** Total number of abnormal pain responses in the control group, prostatitis group, low-dose group, and high-dose group. **(B)** Comparison of the total number of positive reactions in each group on days 20 and 28. Abnormal frequency of pelvic pain response in the control group **(C)**, prostatitis group **(D)**, low-dose group **(E)**, and high-dose group **(F)** under different Von Frey fiber stiffnesses. Differences between groups were compared by a one-factor ANOVA. Data are expressed as mean ± SD. *Significant difference compared with the Control group or prostatitis group, *p < 0.05; ***p < 0.001; ****p < 0.0001; “ns” indicates p > 0.05.

To assess the progression of chronic pelvic pain, we measured tactile allodynia in the lower abdomen adjacent to the prostate on days 0, 20, 22, 24, 26, and 28. Our findings indicate that as the stiffness of the Von Frey fibers increased, the response frequency in the control group remained low (Figure 3C). In contrast, the prostatitis group exhibited consistently high pain response frequencies (Figure 3D). The frequency of pain responses in the low-dose group gradually decreased (Figure 3E), whereas the high-dose group initially showed an increase in pain responses, followed by a gradual decrease (Figure 3F). On the eighth-day post-treatment, the total number of pain reactions in both the low-dose and high-dose groups differed significantly from that observed in the prostatitis group (Figures 3A, B).

### 3.4 Effects of RTX on the central nervous system

**FIGURE 4:**
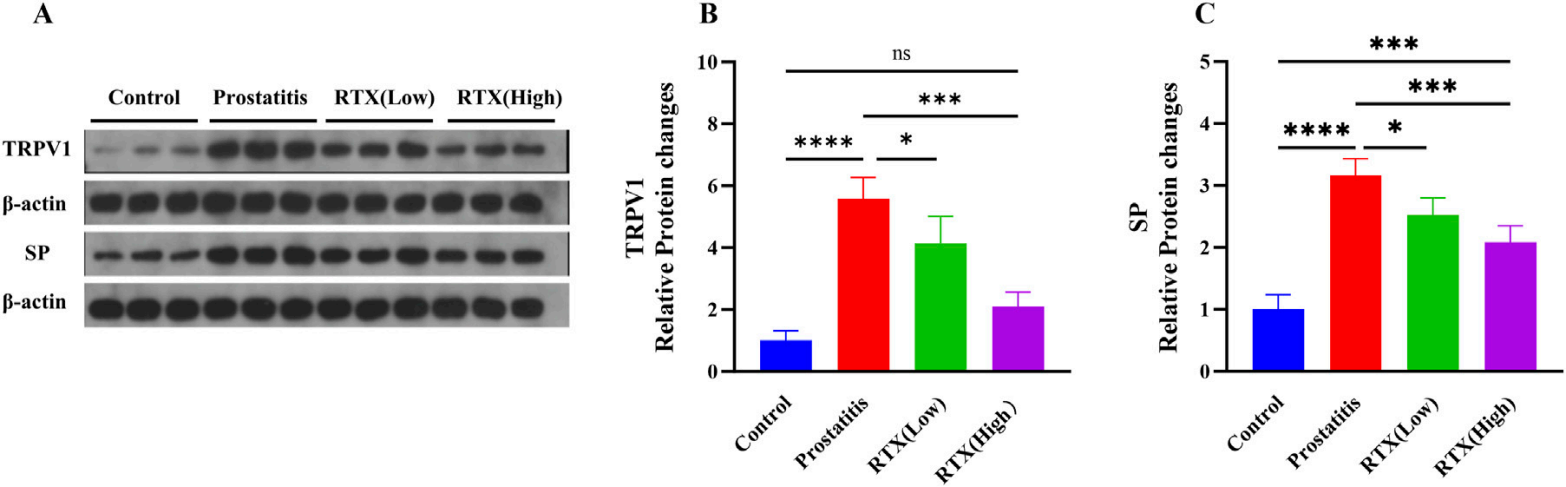
Expression of TRPV1 and SP. **(A)** The expression of TRPV1 in the L6-S1 DRG and the expression of SP in the L6-S1 spinal cord. Statistical results analysis of TRPV1 in the L6-S1 DRG **(B)** and SP in the L6-S1 spinal cord **(C)** between groups. Differences between groups were compared by a one-factor ANOVA. Data represents the mean ± SD, *Significant difference compared with the Control group or prostatitis group, *p < 0.05; ***p < 0.001; ****p < 0.0001; “ns” indicates p > 0.05.

Following prostatic inflammation, there was an observed increase in the expression of TRPV1 in the L6-S1 DRG; however, treatment with RTX resulted in a decrease in TRPV1 expression in the DRG (Figures 4A, B). This reduction may be attributed to RTX’s ability to diminish the sensitivity of the DRG during sensory afferent processing. Secondly, we detected the expression of SP in the spinal cord and observed a decrease in SP expression following RTX treatment (Figures 4A, C). We propose that RTX may influence the central nervous system by modulating the expression of related substances, thereby diminishing the sensitivity of the central sensory system and exerting an analgesic effect.

### 3.5 Effects of RTX on the peripheral sensory nervous system

**FIGURE 5:**
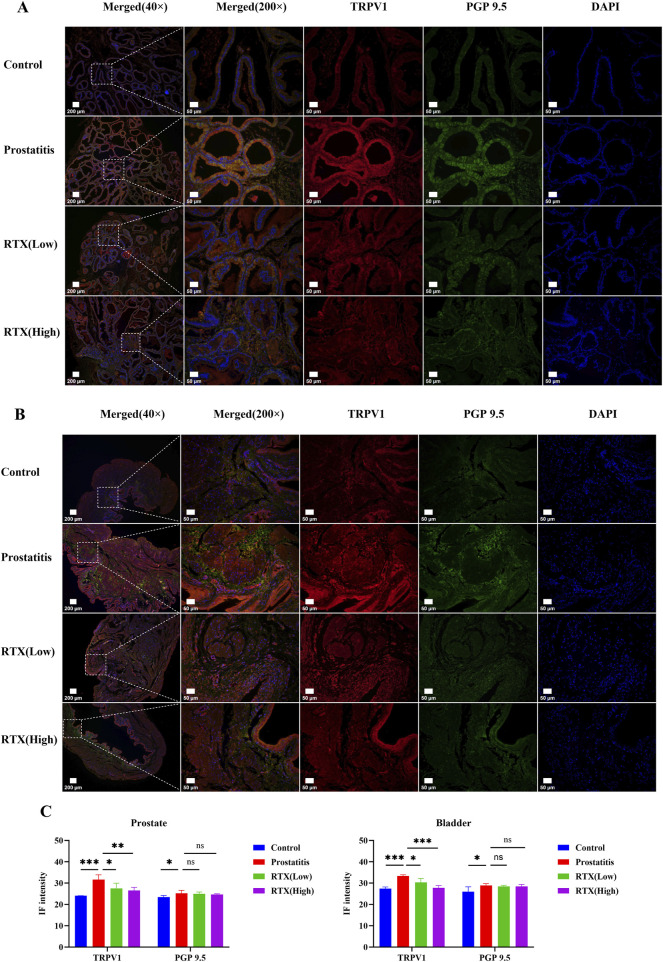
Immunofluorescence analysis of prostate and bladder tissues. **(A)** Immunofluorescence imaging of sensory innervation in the prostate. **(B)** Immunofluorescence imaging of sensory innervation in the bladder. **(C)** Immunofluorescence intensity analysis of prostate and bladder tissue. Differences between groups were compared by a one-factor ANOVA. Data are expressed as mean ± SD. *Significant difference compared with the Control group, *p < 0.05; **p < 0.01; ***p < 0.001; “ns” indicates p > 0.05.

RTX are known to exert their pharmacological effects by desensitizing sensory C-fiber nerves. Consequently, we investigated the innervation of the prostate and bladder. We labeled total nerve fibers with protein gene product 9.5 (PGP 9.5) (Grol et al., 2008) and sensory nerve fibers with TRPV1 (Heng et al., 2011). Our immunofluorescence results indicated that the density of total innervation nerves and sensory afferent nerves in the prostate increased following prostate inflammation. Following RTX treatment, there was no significant change in the total nerve fiber density (Figures 5A, C); however, the density of sensory afferent nerves showed a decrease. A similar pattern was observed in bladder tissue (Figures 5B, C). We speculate that prostatic inflammation primarily leads to an increase in the density of sensory nerves in the prostate and bladder. The administration of RTX may desensitize these sensory nerves in the prostate, thereby diminishing sensory input signals and reducing sensitivity and pain between the organs. Additionally, RTX can also act on the bladder’s sensory afferent nerves to alleviate bladder overactivity.

## 4 Discussion

Chronic prostatitis/chronic pelvic pain syndrome is a multifactorial, chronic inflammatory condition primarily characterized by pain and lower urinary tract symptoms. Nevertheless, the treatment options currently available are limited for many patients. As a typical nociceptive ion channel receptor, TRPV1 is predominantly expressed in neural tissue and plays a crucial role in thermal perception, nociception, inflammation, vasodilation, and contraction (Maximiano et al., 2024). Numerous studies have demonstrated that TRPV1 plays a significant role in the development of CP/CPPS (Jiang et al., 2024). However, there is a paucity of research examining the therapeutic potential of TRPV1 in the context of prostatitis. Consequently, our study investigates the role of TRPV1 in the progression of prostatitis and evaluates whether desensitization of this channel may serve as a beneficial treatment for alleviating symptoms associated with CP/CPPS.

RTX is a potent analog of capsaicin that induces sensory nerve sensitization by binding to TRPV1 receptors, subsequently leading to the desensitization of sensory nerves. These desensitized sensory nerves become unresponsive to various stimuli and do not react to sensory input signals, thereby exerting a long-lasting analgesic effect (Jeffry et al., 2009; Iadarola and Mannes, 2011; Brown, 2016). Research indicates that upon binding to the TRPV1 receptor, RTX elicits three biological effects: excitation, desensitization, and neurotoxicity (Szallasi and Blumberg, 1999). The analgesic effect leverages the long-term non-stress response resulting from excessive activation of this receptor. In the study conducted by Javed (Javed et al., 2022) et al., neuropathic pain resulting from nerve damage was completely alleviated through the administration of RTX via skin injection. Furthermore, research by Ossipov (Ossipov et al., 1999) et al. demonstrated that subcutaneous injection of RTX alleviates neuropathic pain by inducing desensitization of C-fibers. In this study, we observed elevated expression levels of TRPV1 and PGP9.5, as determined by immunofluorescence techniques, along with heightened pain sensitivity measured using the von Frey test, following prostatic inflammation. Conversely, treatment with RTX resulted in a significant decrease in both their expression levels and pain sensitivity. These findings suggest that RTX exerts an analgesic effect by desensitizing the sensory nerve fibers in the prostate, thereby reducing the transmission of pain signals. In addition to pain symptoms, LUTS significantly impact the quality of life of patients with chronic prostatitis. Relevant studies have demonstrated that prostatitis may result in abnormal activation of bladder sensory nerves, contributing to symptoms of frequent urination (Schwartz et al., 2016; Park et al., 2018). Schwartz et al. (2016) study demonstrated that a DRG co-innervates both the prostate and bladder. Furthermore, inflammatory changes in the prostate can sensitize the afferent pelvic nerve (PN) that innervates the bladder. Subsequent research by Park et al. (2018) indicated that functional alterations in the bladder may result from non-bacterial prostatitis, which activates interneurons in the bladder and preganglionic neurons (PGN) in the lumbosacral spinal cord. In our study, we observed that prostatitis increases the density of bladder sensory nerve fibers, which in turn exacerbates symptoms of frequent urination. Following treatment with RTX, there was a significant improvement in these symptoms. The results of this study suggest that RTX has the potential to modulate the abnormal activation of bladder nerves induced by prostatitis, thereby alleviating bladder overactivity associated with this condition.

The expression of TRPV1 in the DRG is associated with organ cross-sensitization (Zhang et al., 2019b), which contributes to the exacerbation of symptoms in patients with CP/CPPS. Numerous animal studies have demonstrated that TRPV1 in the L6-S1 DRG plays a significant role in the sensitization of visceral organs in chronic prostatitis (Funahashi et al., 2019; Zhang et al., 2019a; Roman et al., 2020). Furthermore, the heightened activity of TRPV1 in the DRG contributes to the subsequent development of pain sensitization (Zhang et al., 2007; Srinivasan et al., 2008). Our study demonstrated a significant decrease in TRPV1 expression in the L6-S1 DRG following treatment with RTX. This finding indicates that RTX effectively diminishes the excitability of DRG neurons through desensitization, subsequently leading to a reduction in the cross-sensitization of internal organs. After further analyzing the effects of different doses of RTX, we found that the high-dose RTX group significantly reduced the expression level of TRPV1 in the L6-S1 DRG compared to the low-dose RTX group. Additionally, the pain sensitivity in the RTX treatment group was lower than that in the prostatitis group; however, the pain sensitivity of the high-dose RTX group was higher than that of the low-dose RTX group. We speculate that during the treatment period (days 20–28), large doses of RTX will rapidly sensitize TRPV1 receptors, rendering them responsive to a variety of molecules. Following a brief sensitization period, TRPV1 receptors are gradually depleted, which manifests as receptor defunctionalization and desensitization. This process is characterized by an initial sharp increase in pain sensitivity, followed by a gradual decrease. In contrast, the low-dose RTX group exhibits minimal sensitization during treatment, primarily demonstrating a slow desensitization of TRPV1 receptors, with pain sensitivity declining at a slower rate. However, given the brevity of our observation period following RTX treatment, it may not adequately capture the long-term changes in pain sensitivity. Notably, we discovered that low-dose RTX is associated with a shorter sensitization period and improved safety profile. These findings may offer significant advantages for clinical applications and support the advancement of new strategies aimed at enhancing the safety of treatments for CP/CPPS.

In addition to its role in peripheral sensory nerves, RTX also has a significant role in the central nervous system that warrants attention. SP is a crucial neurotransmitter in the central nervous system, responsible for amplifying and sustaining pain signals (Humes et al., 2024). In the study conducted by Zhang et al. (2009), it was discovered that the persistence and severity of prostate pain correlate with elevated expression levels of the excitatory transmitter SP and its receptors in the spinal cord. Furthermore, subsequent research by Tang et al. (2007) demonstrated that intrathecal injection of RTX leads to a reduction in SP expression within the spinal cord of rats suffering from prostatitis. These studies indicate that SP plays a critical role in central pain mechanisms associated with prostatic inflammation. Our research findings support this conclusion. Through Western blotting experiments, we observed a significant decrease in the expression of SP in the spinal cord of rats with prostatic inflammation following RTX treatment. These results indicate that RTX may exert its analgesic effects by diminishing signaling in the central nervous system.

Due to the widespread distribution of RTX receptors throughout the body, subcutaneous injection of RTX results in systemic drug action (Kissin and Szallasi, 2011). This can provide therapeutic benefits for prostatitis; however, it may also lead to certain side effects. In this study, we observed that a few minutes post-injection of RTX solution without anesthesia, the rats in the RTX treatment group exhibited a gradual increase in body temperature and mild respiratory depression compared to the prostatitis group. Notably, these responses were more pronounced in the high-dose RTX group and returned to normal levels within hours. Consequently, when administering RTX for treatment, it is essential to meticulously regulate the drug concentration to prevent severe respiratory depression and elevated body temperature resulting from excessively high concentrations, as well as to avoid inadequate desensitization effects that may arise from concentrations that are too low. Future research should further investigate the ‘therapeutic window’ of RTX to ascertain its optimal therapeutic concentration and dosage.

While this study offers valuable insights into the mechanism of action by assessing the effects of various doses of RTX in the treatment of CP/CPPS, it also presents certain limitations. First of all, this study primarily focused on the role of RTX in sensory nerves and the central nervous system, without an in-depth exploration of the specific mechanisms underlying RTX’s effects on the nervous system. Furthermore, although RTX demonstrated significant efficacy in this experiment, its long-term safety warrants further investigation. Future research should investigate the long-term effects of RTX in the treatment of chronic prostatitis and its potential clinical applications. Additionally, it is essential to gain a deeper understanding of the role of RTX in both the central and peripheral nervous systems, as well as its influence on organ sensitization resulting from chronic prostatitis.

## 5 Conclusion

The results of this study indicate that: 1) Formalin-induced prostatitis can mimic urinary and pain symptoms observed in patients with chronic prostatitis. 2) RTX diminishes both sensory input and output signals by desensitizing sensory nerve C fibers, thereby exerting its pharmacological effects. 3) RTX decreases the sensitivity of DRG neurons by downregulating the expression of TRPV1, which in turn diminishes inter-organ sensitivity. Additionally, by acting on the central nervous system, RTX reduces the expression of the pain-inducing SP, thereby producing an analgesic effect. 4) Various doses of RTX can effectively treat chronic prostatitis; however, low-dose RTX (200 μg/kg) demonstrates a brief sensitization period and favorable safety profile.

## Data Availability

The raw data supporting the conclusions of this article will be made available by the authors, without undue reservation.
