# Supplementary figures and images for: Targeting TRPV1 channels in desensitized neural afferent pathways may help mitigate pain and lower urinary tract symptoms caused by prostatitis
Source: Front Pharmacol. 2025 Feb 25;16:1541684. doi: 10.3389/fphar.2025.1541684 (PMC11893827; doi:10.3389/fphar.2025.1541684)

## SP in the L6-S1 spinal cord

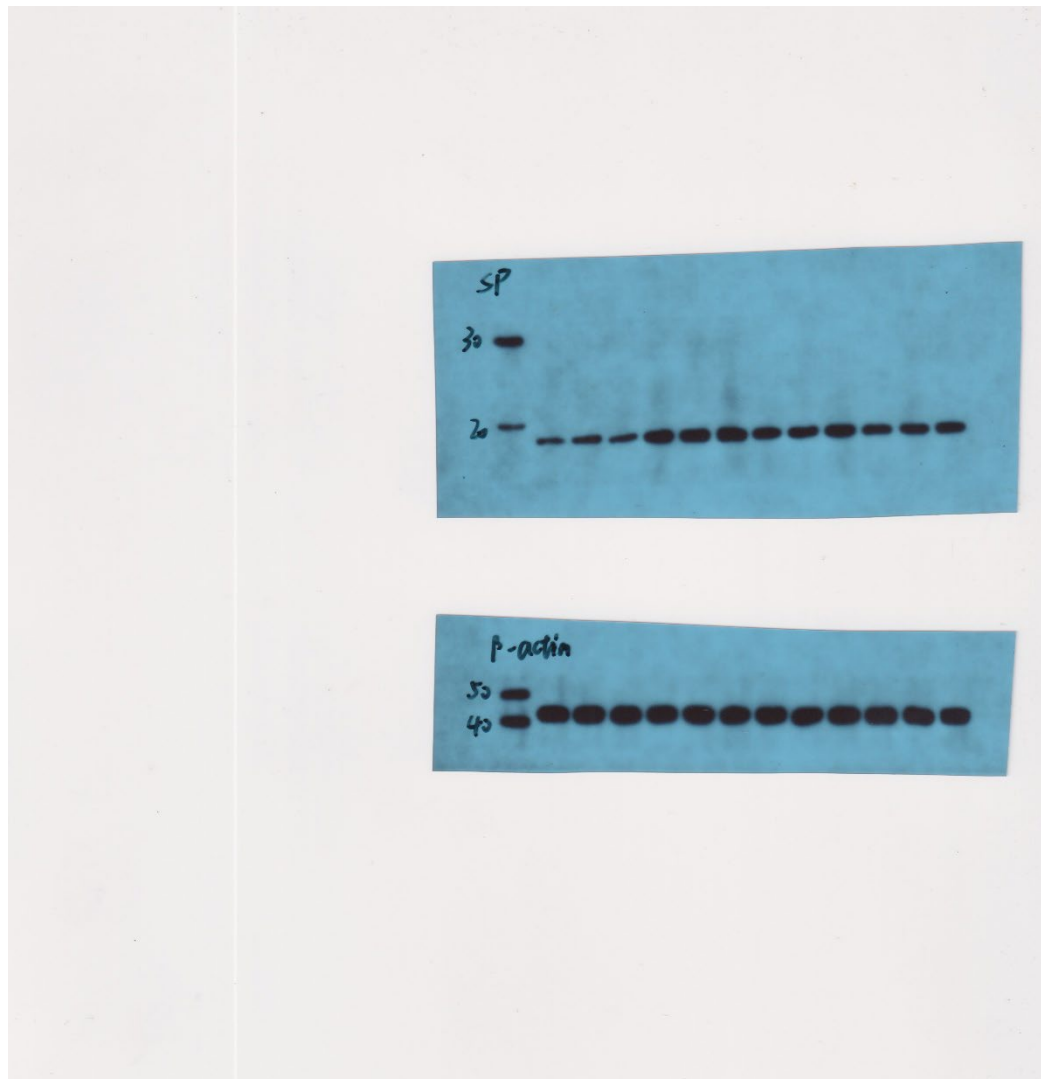

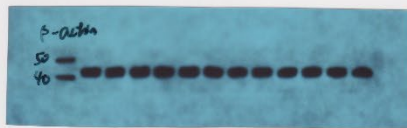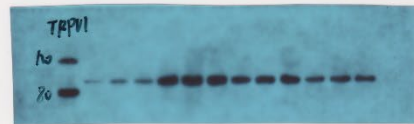

Supplement: Supplementary file 1 [file Image1.pdf]
